# Misperception of poor asthma control in the outpatients clinic of a tertiary hospital in Rio de Janeiro

**DOI:** 10.31744/einstein_journal/2021AO6259

**Published:** 2021-12-20

**Authors:** Carlos Leonardo Carvalho Pessôa, Isabella Araujo Martins, Gustavo Gomes Rodrigues, Isaías José de Carvalho, Elaini Aparecida de Oliveira, Roberta Freitas Momenté, Leticia Vassuler Baldon

**Affiliations:** 1 Universidade Federal Fluminense Niterói RJ Brazil Universidade Federal Fluminense, Niterói, RJ, Brazil.

**Keywords:** Asthma, Surveys and questionnaires, Perception, Control, Signs and symptoms

## Abstract

**Objective:**

To determine the proportion of patients with asthma with misperception of poor control of their disease.

**Methods:**

A cross-sectional study with a convenience sample of patients with asthma and aged ≥18 years. Asthma control was assessed by the Asthma Control Test and the Global Initiative for Asthma questionnaire. The Kappa coefficient was used to analyze the agreement between the results of these tests and the patients’ perception of asthma control, defined by the response to one question of the Asthma Control Test: “How do you evaluate your asthma control during the last 4 weeks?”.

**Results:**

Among the 71 patients aged 19 to 81 years and a mean of 57.7±13.9 years, there were 27 (38%) controlled, according to the Asthma Control Test, and 18 (25.3%) using the Global Initiative for Asthma questionnaire. The Kappa coefficients of the results of these tests and the perception of control by the patients were 0.4 and 0.29, respectively. Among the 41 (57.7%) patients who considered themselves controlled, 18 (43.9%) had a misperception of their poor control, as per the Asthma Control Test, and 25 (61%) by the Global Initiative for Asthma.

**Conclusion:**

Applying the Asthma Control Test, it was observed that almost half of the participants had a misperception of their poor control of the disease and, according to the Global Initiative for Asthma questionnaire, more than half of the sample did not notice the lack of asthma control.

## INTRODUCTION

Asthma affects approximately 300 million people worldwide.^(1)^ In Brazil, in 2018, there were 2,270 deaths by asthma, that is, more than six per day.^(2)^

The goals of asthma treatment are to control symptoms and reduce future risk.^(3,4)^ However, in Brazil and worldwide, most individuals who suffer from asthma do not have their disease under control.^(5,6)^

There is evidence that about half of the patients with severe asthma symptoms consider that their disease is well-controlled.^(7)^

Asthma patients with misperception of the control of their disease are at greater risk of underestimating it and receiving insufficient treatment,^(8)^ with greater risk of exacerbations.^(9)^ In addition, due to inadequate assessment, physicians can overestimate the levels of control, or the extent of improvement achieved with therapy.^(10)^ Awareness of the level of control of the disease on the part of the patient helps in titration of their treatment.

## OBJECTIVE

To determine the proportion of asthma patients with misperception of poor control of their disease.

## METHODS

This is a cross-sectional study with a convenience sample, whose inclusion criteria were age ≥18 years, confirmed diagnosis of asthma according to the criteria of the Global Initiative for Asthma (GINA) document,^(4)^ not being in exacerbation, and not being in the first consultation at the asthma outpatient clinic of the *Hospital Universitário Antônio Pedro* of the *Universidade Federal Fluminense* (UFF). From March 2017 to March 2018, on the days of their pre-scheduled appointments, patients were asked to complete a questionnaire with items on sociodemographic data regarding the level of disease control. Also assessed was the severity of obstructive ventilatory disorder with the most current spirometry. The research protocol was approved by the local Ethics Committee under CAAE: 56248816.1.0000.5243 and opinion no. 3.304.939. The study participants signed the Informed Consent Form (ICF).

The level of asthma control was assessed by means of two questionnaires (Table 1). The Asthma Control Test (ACT)^(11)^consists of five questions that address limitations caused by asthma, presence of dyspnea, nighttime awakenings due to the disease, need for relief medication, and self-assessment of asthma control. Its evaluation is restricted to the last 4 weeks. Each question presents five alternatives, which generate scores of one to five. The maximum score is 25 points and the minimum, is five points. In this study, asthma was considered controlled when scores ≥20 were obtained, a value used as a control marker in most studies, including the one that gave rise to the ACT. The questionnaire proposed by GINA^(4)^ has four questions similar to those of ACT, with only two response alternatives (yes or no). Each question is worth one point, so that the maximum score is four. Patients are considered controlled only when they do not score on this questionnaire. There is no question in GINA similar to the fifth ACT question about patient self-assessment of asthma control.

**Table 1:** Questions and scores from Asthma Control Test and Global Initiative for Asthma

ACT	GINA
1. Has asthma hindered your activities at work, school, or home? Not at all (5) A few times (4) Sometimes (3) Most of the time (2) All the time (1)	1. Any activity limitation? Yes (1) No (0)
2. How many times have you been shortness of breath? Never (5) 1 or 2 times a week (4) 3 to 6 times a week (3) Once a day (2) More than once a day (1)	2. Daytime symptoms >2 times a week? Yes (1) No (0)
3. Did asthma wake you up at night or earlier than usual in the morning? Not at all (5) 1 or 2 times (4) 1 time a week (3) 2 or 3 nights a week (2) 4 or more nights a week (1)	3. Any nighttime awakenings due to asthma? Yes (1) No (0)
4. How many times have you used the medicine by inhalation, nebulization for relief? Never (5) 1 time a week or less (4) A few times a week (3) 1 or 2 times a day (2) 3 or more times a week (1)	4. Need for rescue medication >2 times per week? Yes (1) No (0)
5. How do you rate the control of your asthma? Completely controlled (5) Well-controlled (4) Somewhat controlled (3) Poorly controlled (2) Totally uncontrolled (1)	
Controlled: ≥20 points Uncontrolled: <20 points	Controlled: score = 0 Partially controlled: 1 or 2 points Uncontrolled: 3 or 4 points

Source: Adapted from Global Initiative for Asthma (GINA). Global strategy for asthma management and prevention: updated 2018. Fontana: GINA; 2018 [cited 2020 Apr 30]. Available from: https://ginasthma.org/wp-content/uploads/ 2019/01/2018-GINA.pdf;^(4)^ Nathan RA, Sorkness CA, Kosinski M, Schatz M, Li JT, Marcus P, et al. Development of the asthma control test: a survey for assessing asthma control. J Allergy Clin Immunol. 2004;113(1):59-65.^(11)^

ACT: Asthma Control Test; GINA: Global Initiative for Asthma.

**Figure 1:**
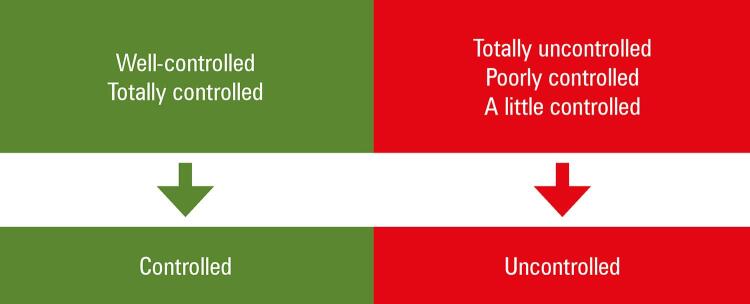
Division of patients, according to answer to the question from the Asthma Control Test “How do you evaluate the control of your asthma?”

Analysis of the patient’s perception of asthma control was performed based on the following question in the ACT: “How do you rate your asthma control during the last 4 weeks?” The response options offered to the question and the division of the groups can be seen in figure 1.

We investigated the agreement between the answer to this key question and the level of asthma control by means of the total score obtained in the ACT and in the GINA questionnaire.

For the descriptive analysis of the numerical data, measures of central tendency and dispersion were used. Calculations of simple and relative frequencies and percentages were used for the questions with categorized answers. Associations of categorical variables were verified using the statistical χ^2^ test or Fisher’s exact test, as theoretically indicated. Values of p<0.05 indicated statistical significance. We analyzed the agreement between the perception of asthma control according to the patient and the level of control in ACT and GINA by means of the Kappa coefficient, which classifies the agreement as slight (0.01 to 0.2), fair (0.21 to 0.4), moderate (0.41 to 0.6), substantial (0.61 to 0.8), and almost perfect (0.81 to 0.99). The data obtained were digitized on a Microsoft Excel 2010 spreadsheet and imported for statistical analysis at the Epi Info™ 7.2 software.

## RESULTS

A total of 71 participants between 19 and 81 years of age, mean age of 57.7±13.9 years, were included. Fifty (70.4%) were nonsmokers, four (5.6%) smokers, and 17 (24%) former smokers. Regarding severity of the obstructive disorder, 11 (15.5%) had normal spirometry, 39 (54.9%) had mild obstructions, 13 (18.3%) had moderate obstructions, and six (8.4%) had severe obstructions; two patients (2.8%) could not reliably perform the test. Patients with more severe obstructions, as well as those who were unmarried, were more often found to be uncontrolled by the ACT, a finding not corroborated by GINA. According to the 2018 GINA document, two participants (2.8%) were in stage 1 of asthma treatment (short-acting beta2-agonist as needed), 14 (19.7%) in stage 2 (low-dose inhaled corticosteroids), 11 (15.5%) in stage 3 (low-dose inhaled corticosteroid and long-acting bronchodilator), 43 (60.6%) in stage 4 (moderate or high dose of inhaled corticosteroids and long-acting bronchodilator), and one (1.4%) in stage 5 (oral corticosteroids used regularly and/or anti-immunoglobulin E - IgE, associated with a combination of inhaled corticosteroids and bronchodilators). Twenty-seven patients (38.0%) had controlled asthma according to ACT, and 18 (25.3%) according to GINA. Table 2 shows the associations of sociodemographic and clinical characteristics with asthma control according to ACT and GINA.

**Table 2:** Association of sociodemographic and clinical characteristics with asthma control according to the Asthma Control Test and Global Initiative for Asthma

Variable	Total	ACT			GINA		
	
		Controlled	Uncontrolled	p value	Controlled	Uncontrolled	p value
Sex				0.4			0.49
Female	61 (85.9)	22 (31.0)	39 (54.9)		15 (21.1)	46 (64.8)	
Male	10 (14.1)	5 (7.0)	5 (7.0)		3 (4.2)	7 (9.9)	
Marital status				0.038			0.23
Married	31 (43.7)	16 (22.5)	15 (21.1)		10 (14.1)	21 (29.6)	
Unmarried	40 (56.3)	11 (15.5)	29 (40.8)		8 (11.2)	32 (45.1)	
Level of education*				0.6			0.19
Up to 9 years	37 (52.1)	13 (18.3)	24 (33.8)		7 (9.9)	30 (42.2)	
More than 9 years	34 (47.9)	14 (19.7)	20 (28.2)		11 (15.5)	23 (32.4)	
Personal income^†^				0.9			0.07
Up to 1 MW	49 (70.0)	18 (25.7)	31 (44.3)		9 (12.9)	40 (57.1)	
> 1 MW	21 (30.0)	8 (11.4)	13 (11.4)		8 (11.4)	13 (18.6)	
Family income				0.22			0.09
Up to 1 MW	28 (40.0)	8 (11.4)	20 (28.6)		4 (5.7)	24 (34.3)	
> 1 MW	42 (60.0)	18 (25.7)	24 (34.3)		13 (18.6)	29 (41.4)	
Age, years				0.26			0.054
≥70	16 (22.5)	8 (11.3)	8 (11.3)		7 (9.8)	11 (15.5)	
<70	55 (77.5)	19 (26.8)	36 (50.7)		9 (12.7)	44 (62.0)	
Obstruction				0.018			0.28
Mild/absent	50 (72.5)	23 (33.3)	27 (39.1)		14 (20.3)	34 (49.3)	
Moderate/severe	19 (27.5)	3 (4.3)	16 (23.2)		4 (5.8)	17 (24.6)	
Smoker				0.1			0.16
No/former smoker	50 (70.5)	22 (31.0)	28 (39.5)		15 (21.1)	35 (49.3)	
Yes	21 (29.5)	5 (7.0)	16 (22.5)		3 (4.2)	18 (25.4)	

Results expressed by n (%).

* Level of education - complete or not; ^†^ The Brazilian [monthly] MW on September 27, 2020, was U$ 184.00.

ACT: Asthma Control Test; GINA: Global Initiative for Asthma; MW: minimum wage.

Table 3 shows the distribution of the sample according to answers to the key question of the study, and the agreement of each item with the ACT and GINA. There was agreement between the patient’s opinion on asthma control and the ACT in 49 (69.0%) cases; the Kappa coefficient between the patient’s perception of asthma control and the level of control according to the ACT was 0.4, considered fair. There was agreement in 44 (62.0%) cases between the patient’s opinion and the questionnaire proposed by GINA. The Kappa coefficient was 0.29, and also considered fair. Among the 34 participants who marked the well-controlled option on the key question of the study, 22 (64.7%) were uncontrolled according to the GINA document, and 17 (50%) were uncontrolled on the ACT.

**Table 3:** Distribution of the sample according to self-assessments* of asthma control and agreement on the level of control in the Asthma Control Test and the Global Initiative for Asthma

Patient’s perception	ACT				GINA			
	
	Controlled asthma	Uncontrolled asthma	Total	Agreement	Controlled asthma	Uncontrolled asthma	Total	Agreement
Not controlled at all	0	2 (2.8)	2 (2.8)	100	0	2 (2.8)	2 (2.8)	100
Poorly controlled	0	7 (9.9)	7 (9.9)	100	0	7 (9.9)	7 (9.9)	100
Somewhat controlled	4 (5.6)	17 (23.9)	21 (29.5)	81	2 (2.8)	19 (26.8)	21 (29.6)	90.5
Well-controlled	17 (23.9)	17 (23.9)	34 (47.9)	50	12 (16.9)	22 (31.0)	34 (47.9)	35.3
Completely controlled	6 (8.4)	1 (1.4)	7 (9.9)	85.7	4 (5.6)	3 (4.2)	7 (9.8)	57.1
Total	27 (38.0)	44 (62.0)	71 (100)	69	18 (25.3)	53 (74.7)	71 (100)	62

Results expressed by n (%) or only %.

* Defined by the answer options selected by the participants for one of the ACT questions, shown in the first column on the left. Controlled individuals marked options well-controlled, or completely controlled. Uncontrolled participants checked somewhat controlled, poorly controlled, or totally uncontrolled.

ACT: Asthma Control Test; GINA: Global Initiative for Asthma.

In table 4, the sample was separated into controlled (those who marked the key question as totally controlled or well-controlled) and uncontrolled (those who marked totally uncontrolled, poorly controlled, or somewhat controlled). We found that with ACT, 43.9% of uncontrolled patients perceived lack of asthma control, and with GINA, this percentage increased to 61%. Considering the misperception of the level of control in the entire sample, 67% inappropriately perceived their level of control.

**Table 4:** Patient perception of asthma control on the Asthma Control Test and the Global Initiative for Asthma

Perception of control by the patient*	ACT			GINA			Total
	
	Controlled	Uncontrolled	Misperception	Controlled	Uncontrolled	Misperception	
Controlled	23 (32.5)	18 (25.3)	43.9	16 (22.5)	25 (35.2)	61.0	41 (47.7)
Uncontrolled	4 (5.6)	26 (36.5)	13.3	2 (2.8)	28 (39.5)	6.7	30 (42.2)
Total	27 (38.1)	44 (61.9)	57.2	18 (25.3)	53 (74.7)	67.7	71 (100)

Results expressed by n (%) or only %.

* Defined by the response to the ACT question, “How do you rate your asthma control during the past 4 weeks?” Controlled patients checked well-controlled or completely controlled. Uncontrolled patients checked somewhat controlled, poorly controlled, or totally uncontrolled.

ACT: Asthma Control Test; GINA: Global Initiative for Asthma.

Table 5 shows the perception of control had no statistically significant association with the sociodemographic and clinical profile, regardless of the questionnaire used, GINA or ACT.

**Table 5:** Association of quality of asthma perceived control with sociodemographic and clinical characteristics

Variable	Perception of control by ACT*				Perception of control by GINA*			
	
	Total	Correct	Incorrect	p value	Total	Correct	Incorrect	p value
Sex				0.46				0.6
Female	57 (85.1)	41 (61.2)	16 (23.9)		59 (85.5)	36 (52.2)	23 (33.3)	
Male	10 (14.9)	8 (11.9)	2 (3.0)		10 (14.5)	6 (8.7)	4 (5.8)	
Marital status				0.66				0.53
Married	29 (43.3)	22 (32.8)	7 (10.5)		30 (43.5)	17 (24.6)	13 (18.9)	
Unmarried	38 (56.7)	27 (40.3)	11 (16.4)		39 (56.5)	25 (36.2)	14 (20.3)	
Level of education^†^				0.44				0.96
Up to 9 years	35 (52.2)	27 (40.3)	8 (11.9)		36 (52.1)	22 (31.8)	14 (20.3)	
More than 9 years	32 (47.8)	22 (32.8)	10 (14.9)		33 (47.9)	20 (29.0)	13 (18.9)	
Personal income^‡^				0.78				0.1
Up to 1 MW	46 (69.7)	33 (50.0)	13 (19.7)		28 (70.6)	26 (38.2)	22 (32.4)	
>1 MW	20 (30.3)	15 (22.7)	5 (7.6)		20 (29.4)	15 (22.0)	5 (7.4)	
Family income				0.44				0.14
Up to 1 MW	28 (47.8)	19 (28.8)	9 (13.6)		28 (41.2)	14 (20.6)	14 (20.6)	
>1 MW	38 (52.8)	29 (44.0)	9 (13.6)		40 (58.8)	27 (39.7)	13 (19.1)	
Age, years				0.98				0.46
≥70	15 (22.4)	11 (16.4)	4 (7.5)		16 (23.2)	11 (15.9)	5 (7.3)	
<70	52 (77.6)	38 (56.7)	14 (20.9)		53 (76.8)	31 (44.9)	22 (31.9)	
Obstruction				0.28				0.65
Mild /absent	45 (69.2)	35 (53.8)	10 (15.4)		36 (68.7)	28 (41.8)	18 (26.9)	
Moderate/severe	20 (30.8)	13 (20.0)	7 (10.8)		21 (31.3)	14 (20.9)	7 (10.4)	
Smoker				0.9				0.32
No/former smoker	48 (71.6)	35 (52.2)	13 (19.4)		49 (68.7)	28 (40.6)	21 (30.4)	
Yes	19 (28.4)	14 (20.9)	5 (7.5)		20 (31.3)	14 (20.3)	6 (8.7)	

* Defined by response to ACT: “How do you rate the control of your asthma over the past 4 weeks?” Controlled checked well-controlled or completely controlled options. Uncontrolled checked somewhat controlled, poorly controlled, or totally uncontrolled; ^†^ education completed or not; ^‡^ the Brazilian [monthly] MW on September 27, 2020 was U$ 184.00.

ACT: Asthma Control Test; GINA: Global Initiative for Asthma; MW: minimum wage.

## DISCUSSION

In this service, inhalation technique is demonstrated to all patients at every visit. Patients are invariably asked to bring their empty inhaler devices for training at every visit, and, when they do, they perform the inhalation technique in front of the physician; when they do not, the physicians always demonstrate the technique with an empty device identical to the one used by each patient. If mistakes are found, they are always corrected.^(12)^ In addition, the main asthma-related comorbidities, such as rhinitis, gastroesophageal reflux, anxiety, depression, and smoking, are investigated and treated at the outpatient clinic. Maybe these behaviors are the reasons for 25% to 38% of the sample showing controlled asthma. If it is still less the ideal scenario, it is higher than 9% and 13% observed in recent national studies,^(5,13)^despite the fact the most highly complex cases were managed at this tertiary hospital outpatient clinic. When using the ACT, no associations were observed between uncontrolled and low income or low education level, as previously demonstrated.^(14)^ On the other hand, it was found that unmarried patients and those with more severe obstructions were more often uncontrolled, suggesting that special attention should be given to these groups. On the other hand, the GINA did not show associations between clinical, sociodemographic, or functional characteristics of the sample and asthma control, suggesting that supervision of the level of control should be universal.

Poorly-controlled or incorrectly treated asthma can progress unfavorably with near-fatal exacerbations and even result in death.^(15)^ Up to 66% of patients with asthma did not spontaneously report their symptoms to their physician,^(16)^ and approximately 50% with severe persistent symptoms considered their disease well-controlled.^(17)^ Patient´s misperception of asthma control can lead to inappropriate assessment of the disease by the physician and, consequently, to insufficient or excessive treatment.^(16)^ There is still no consensus on a gold standard for measuring control objectively.^(12)^ The current GINA document emphasizes the need to assess asthma control to guide asthma management decisions, and proposes the use of a questionnaire, which, although widely recommended and accepted, is not formally validated.^(4)^ The ACT^(11)^ is a validated questionnaire, including in the Portuguese version, and is reproducible and sensitive for the assessment of control.^(18)^ It is one of the tools recommended by the American Thoracic Society (ATS)/European Respiratory Society (ERS) task force on the standardization of asthma control measures.^(19)^ In the present study, 41 (57.7%) perceived themselves to be controlled, whereas, in fact, 18 (43.9%) of individuals were not controlled by ACT, and 25 (61%) were not controlled by GINA. These results are lower than the 68.5% seen in a sample other than that of the present study, since this was a survey conducted in pharmacies,^(20)^and higher than the 27.2% obtained when another questionnaire, the Asthma Control Questionnaire (ACQ-6), was used.^(13)^ It is important to emphasize that half of the patients who specifically marked the well-controlled option on the ACT failed to perceive inadequate asthma control according to the ACT score itself, and, using the GINA, 64.7% of those who marked this option actually did not have the disease controlled. Among the 30 patients who considered their asthma uncontrolled, four (13.3%) were controlled by ACT, and two (6.7%) by GINA. In the latter, the existence of other comorbidities, such as psychiatric disorders, may justify this confusion. In this outpatient clinic, it has been verified that at least 53% of patients have anxiety and/or depression.^(21)^Perhaps these patients have some dissatisfaction with their ventilation due to somatization or hyperinflation, and such could generate mathematical repercussions in the questions and scores in questionnaires. In the total sample, 22 (31%) participants misinterpreted their disease control level when using ACT and 27 (38%) in GINA.

Another aspect to be highlighted is that, unlike other questionnaires, in one of its questions, the ACT allows the patient to make a self-assessment of their control, which is the key question of this study. It has been shown patients with misperception of their poor control often answered this question inadequately, mistakenly marking the totally controlled option or, even more often, the well-controlled option. This increased the score obtained in this question and, consequently, in the final score of the questionnaire, which may have contributed to classifying the patient as controlled on the ACT - although sometimes this is not the reality. Using, *e.g.,* the questionnaire proposed in the GINA document, which does not offer this question for self-assessment, we detected an even higher number of uncontrolled patients, who claimed to be controlled in this series. In any case, the Kappa coefficient between GINA or ACT asthma control, and the perception of control according to the patient was low in both.

What was previously verified was that certain groups perceive themselves inappropriately, such as the elderly,^(22)^ women^(23)^ and patients with asthma considered mild^(21)^ was not confirmed. This may have occurred due to the exclusion in this study of controlled patients with no perception of adequate control, since the current objective is to evaluate only the perception of poor control. Neither the fact of being an outpatient clinic coordinated by a specialist seems to influence the quality of the patients’ perception of control.^(20)^

Among the limitations of this study is the possibility of selection bias of patients who comprised a convenience sample from a teaching hospital, which may not be representative of the general population of outpatients. The patients’ recall of asthma symptoms in the past month may not be completely accurate, and the possibility of recall bias cannot be ruled out. Another limitation is the sample size. Finally, the ACT question used to assess patients’ perception of asthma control “How would you rate your asthma control in the past 4 weeks? was not validated for this purpose.

To our knowledge, using ACT and GINA, this is the only observational study ever conducted to investigate the difficulty of perception of poor asthma control in a tertiary hospital in a real-life study in Brazil. The notion of control seems to be little understood by asthma patients. Perhaps, they are satisfied with partial asthma control and consider symptom persistence acceptable.^(24)^ Patients are often heard saying phrases such as “Doctor, I know my body,” but that perception may prove to be flawed when measured by objective methods. Close attention and rigor are required when assessing asthma control. In addition to investigating treatment adherence, inhaler technique quality, and treatment of comorbidities to achieve asthma control, it is important to remember that misperception of level of control is very common. There are patients who have controlled asthma and consider it uncontrolled, but much more often there are patients who consider themselves controlled without actually being so. Better education is needed regarding the importance, perception, and methods of identifying control. Perhaps familiarizing asthmatic patients with these same questionnaires and orienting them to use them in their homes are good strategies.

## CONCLUSION

The Asthma Control Test showed that almost half of the participants had a misperception of their poor asthma control and, according to the Global Initiative for Asthma questionnaire, more than half of the sample did not perceive the lack of asthma control. Misperception is very common, worrisome, and urges the use of questionnaires in medical practice, for a more reliable assessment of control and correct titration of treatment, regardless of the characteristics of patients.
