# Supplementary material for: Misperception of poor asthma control in the outpatients clinic of a tertiary hospital in Rio de Janeiro
Source: Einstein (Sao Paulo). 2021 Dec 20;19:eAO6259. doi: 10.31744/einstein_journal/2021AO6259 (PMC8693884; doi:10.31744/einstein_journal/2021AO6259)
Supplement: Translation

# Translation: Má percepção da falta de controle da asma em ambulatório de hospital terciário do Rio de Janeiro

**Authors:** Carlos Leonardo Carvalho Pessôa, Isabella Araujo Martins, Gustavo Gomes Rodrigues, Isaías José de Carvalho, Elaini Aparecida de Oliveira, Roberta Freitas Momenté, Leticia Vassuler Baldon

**Affiliations:** 1 Brasil Universidade Federal Fluminense, Niterói, RJ, Brasil.

**Keywords:** Asma, Inquéritos e questionários, Percepção, Controle, Sinais e sintomas

## Abstract

**Objetivo:**

Determinar a proporção de pacientes com asma com percepção inadequada do mau controle de sua doença.

**Métodos:**

Estudo transversal com amostra de conveniência de pacientes com asma e idade ≥18 anos. Avaliou-se o controle da asma por meio do *Asthma Control Test* e do questionário do *Global Initiative for Asthma*. Utilizou-se o coeficiente Kappa para análise da concordância entre os resultados desses testes e a percepção do controle da asma pelo paciente, definida pela resposta a uma questão do *Asthma Control Test*: “Como você avalia o controle da sua asma durante as últimas 4 semanas?”.

**Resultados:**

Entre os 71 pacientes com idades entre 19 e 81 anos e média de 57,7±13,9 anos, existiam 27 (38%) controlados, segundo o *Asthma Control Test*, e 18 (25,3%) de acordo com o questionário do *Global Initiative for Asthma*. Os coeficientes de Kappa entre os resultados desses testes e a percepção do controle pelos pacientes foram, respectivamente, 0,4 e 0,29. Entre os 41 (57,7%) pacientes que se consideravam controlados, 18 (43,9%) tinham percepção inapropriada do seu mau controle, de acordo com o *Asthma Control Test*, e 25 (61%) segundo o *Global Initiative for Asthma*.

**Conclusão:**

Aplicando-se o *Asthma Control Test*, observou-se que quase metade dos participantes tinha percepção inadequada de seu mau controle da doença e, segundo o questionário da *Global Initiative for Asthma*, mais da metade da amostra não percebeu o descontrole da asma.

## INTRODUÇÃO

A asma acomete cerca de 300 milhões de pessoas no mundo.^(1)^ No Brasil, em 2018, ocorreram 2.270 mortes por asma, ou seja, mais de seis por dia.^(2)^

Os objetivos do tratamento da asma são o controle dos sintomas e a redução do risco futuro.^(3,4)^ No entanto, no Brasil e no mundo, a maioria dos portadores de asma não tem sua doença controlada.^(5,6)^

Há evidências de que cerca de metade dos pacientes com sintomas graves de asma considera que sua doença encontra-se bem controlada.^(7)^

Asmáticos com má percepção do controle de sua doença possuem maior risco de subestimá-la e receber tratamento insuficiente,^(8)^com maior risco de exacerbações.^(9)^Além disso, os médicos podem superestimar os níveis de controle ou a extensão da melhora alcançada com a terapia, em razão de uma avaliação inadequada.^(10)^ A ciência do nível de controle da doença, por parte do paciente, auxilia na titulação de seu tratamento.

## OBJETIVO

Determinar a proporção de pacientes portadores de asma com percepção inadequada do mau controle de sua doença.

## MÉTODOS

Trata-se de estudo transversal com amostra de conveniência, cujos critérios de inclusão foram idade ≥18 anos, diagnóstico confirmado de asma segundo os critérios do documento do *Global Initiative for Asthma* (GINA),^(4)^ não se encontrar em exacerbação e não estar em primeira consulta no ambulatório de asma do Hospital Universitário Antônio Pedro da Universidade Federal Fluminense (UFF). No período de março de 2017 a março de 2018, nos dias de suas consultas pré-agendadas, os pacientes foram convidados a preencher um questionário com itens sobre dados sociodemográficos e a respeito do nível de controle da doença. Avaliou-se também a gravidade do distúrbio ventilatório obstrutivo na espirometria mais atual. O protocolo de pesquisa foi aprovado pelo Comitê de Ética local, sob o CAAE: 56248816.1.0000.5243 e parecer 3.304.939. Os participantes do estudo assinaram o Termo de Consentimento Livre e Esclarecido (TCLE).

Avaliou-se o nível de controle da asma por meio de dois questionários (Tabela 1). O *Asthma Control Test* (ACT)^(11)^ é composto de cinco questões que abordam limitações causadas pela asma, presença de dispneia, despertares noturnos em decorrência da doença, necessidade de uso de medicação de alívio e autoavaliação do controle asmático. Sua avaliação se restringe às últimas 4 semanas. Cada questão apresenta cinco alternativas, que geram pontuações de um a cinco. A pontuação máxima é de 25 pontos, e a mínima, cinco pontos. Nesta pesquisa, considerou-se a asma controlada quando foram obtidos escores ≥20, valor utilizado como balizador de controle na maioria dos estudos, inclusive no que deu origem ao ACT. O questionário proposto pelo GINA^(4)^ tem quatro questões semelhantes às do ACT, com apenas duas alternativas de resposta (sim ou não). Pode-se obter um ponto a cada pergunta, de modo que a pontuação máxima é quatro. Pacientes são considerados controlados somente quando não pontuam neste questionário. Não há, no GINA, pergunta similar à quinta questão do ACT sobre autoavaliação do paciente a respeito do controle da asma.

**Tabela 1:** Questões e pontuações do *Asthma Control Test* e da *Global Initiative for Asthma*

ACT	GINA
1. A asma prejudicou suas atividades no trabalho, na escola ou em casa? Nenhuma vez (5) Poucas vezes (4) Algumas vezes (3) Maioria das vezes (2) Todo o tempo (1)	1. Qualquer limitação da atividade? Sim (1) Não (0)
2. Quantas vezes você teve falta de ar? De jeito nenhum (5) 1 ou 2 vezes por semana (4) 3 a 6 vezes por semana (3) 1 vez ao dia (2) Mais que 1 vez ao dia (1)	2. Sintomas diurnos >2 vezes por semana? Sim (1) Não (0)
3. A asma acordou você à noite ou mais cedo que de costume? De jeito nenhum (5) 1 ou 2 vezes (4) 1 vez por semana (3) 2 ou 3 noites por semana (2) 4 ou mais noites por semana (1)	3. Qualquer despertar noturno devido à asma? Sim (1) Não (0)
4. Quantas vezes você usou o remédio por inalação, nebulização para alívio? De jeito nenhum (5) 1 vez por semana ou menos (4) Poucas vezes por semana (3) 1 ou 2 vezes por dia (2) 3 ou mais vezes por semana (1)	4. Necessidade de medicação de resgate >2 vezes por semana? Sim (1) Não (0)
5. Como você avalia o controle da sua asma? Completamente controlada (5) Bem controlada (4) Um pouco controlada (3) Pobremente controlada (2) Totalmente descontrolada (1)	
Controlados: ≥20 pontos Não controlados: <20 pontos	Controlados: pontuação = 0 Parcialmente controlados: 1 ou 2 pontos Não controlados: 3 ou 4 pontos

Fonte: Adaptada de Global Initiative for Asthma (GINA). Global strategy for asthma management and prevention: updated 2018. Fontana: GINA; 2018 [cited 2020 Apr 30]. Available from: https://ginasthma.org/wp-content/uploads/2019/01/2018-GINA.pdf;^(4)^ Nathan RA, Sorkness CA, Kosinski M, Schatz M, Li JT, Marcus P, et al. Development of the asthma control test: a survey for assessing asthma control. J Allergy Clin Immunol. 2004;113(1):59-65.^(11)^

ACT: *Asthma Control Test*; GINA: *Global Initiative for Asthma*.

Foi realizada análise da percepção do controle da asma pelo paciente a partir da seguinte pergunta do próprio ACT: “Como você avalia o controle da sua asma durante as últimas 4 semanas?” As opções de respostas oferecidas à questão e a divisão dos grupos podem ser observadas na figura 1.

**Figura 1:** Divisão dos pacientes, de acordo com a resposta à pergunta do *Asthma Control Test* “Como você avalia o controle da sua asma?”

Investigou-se a concordância entre a resposta a essa pergunta-chave do estudo, presente no ACT e o nível de controle da asma por meio da pontuação total obtida nesse mesmo teste e no questionário do GINA.

Para a análise descritiva dos dados numéricos, utilizaram-se medidas de tendência central e de dispersão. Foram usados os cálculos das frequências simples e relativas e as percentagens para as questões com respostas categorizadas. Verificaram-se as associações das variáveis categóricas utilizando o teste estatístico do χ^2^ ou o teste exato de Fisher, conforme indicação teórica. Valores de p<0,05 indicaram significância estatística. Foi analisada a concordância entre a percepção de controle da asma segundo o paciente e o nível de controle no ACT e no GINA por meio do coeficiente Kappa, que a classifica em pobre (0,01 a 0,2), discreta (0,21 a 0,4), moderada (0,41 a 0,6), substancial (0,61 a 0,8) e quase perfeita (0,81 a 0,99). Os dados obtidos foram digitalizados em planilha do Microsoft Excel 2010 e importados para análise estatística no programa Epi Info™ 7.2.

## RESULTADOS

Foram incluídos 71 participantes entre 19 e 81 anos de idade, com média de 57,7±13,9 anos. Cinquenta (70,4%) eram não tabagistas, quatro (5,6%) tabagistas e 17 (24%) ex-tabagistas. No que se refere à gravidade do distúrbio obstrutivo, 11 (15,5%) apresentavam espirometrias normais, 39 (54,9%) tinham obstruções leves, 13 (18,3%) apresentavam obstruções moderadas, e seis (8,4%) tinham obstruções graves, sendo que dois pacientes (2,8%) não conseguiram realizar o teste de maneira confiável. Pacientes com obstruções mais graves, bem como os que não eram casados, encontravam-se mais frequentemente não controlados pelo ACT, dado não corroborado pelo GINA. Segundo o documento do GINA de 2018, dois participantes (2,8%) encontravam-se no estágio 1 do tratamento da asma (beta2-agonista de curta duração conforme necessidade), 14 (19,7%) no estágio 2 (baixa dose de corticosteroides inalatórios), 11 (15,5%) no estágio 3 (baixa dose de corticosteroide inalatório e broncodilatador de longa ação), 43 (60,6%) no estágio 4 (dose moderada ou alta de corticoide inalatório e broncodilatador de longa ação) e um (1,4%) no estágio 5 (corticosteroides orais usados regularmente e/ou anti-imunoglobulina E – IgE, associados à combinação de corticoides e broncodilatadores inaláveis). Vinte e sete pacientes (38,0%) apresentavam asma controlada segundo o ACT e 18 (25,3%) de acordo com o GINA. A tabela 2 demonstra as associações das características sociodemográficas e clínicas com o controle da asma de acordo com o ACT e o GINA.

**Tabela 2:** Associação das características sociodemográficas e clínicas com o controle da asma de acordo com o *Asthma Control Test* e a *Global Initiative for Asthma*

Variável	Total	ACT			GINA		
	
		Controlado	Não controlado	Valor de p	Controlado	Não controlado	Valor de p
Sexo				0,4			0,49
Feminino	61 (85,9)	22 (31,0)	39 (54,9)		15 (21,1)	46 (64,8)	
Masculino	10 (14,1)	5 (7,0)	5 (7,0)		3 (4,2)	7 (9,9)	
Estado civil				0,038			0,23
Casado	31 (43,7)	16 (22,5)	15 (21,1)		10 (14,1)	21 (29,6)	
Não casado	40 (56,3)	11 (15,5)	29 (40,8)		8 (11,2)	32 (45,1)	
Grau de instrução*				0,6			0,19
Até 9 anos	37 (52,1)	13 (18,3)	24 (33,8)		7 (9,9)	30 (42,2)	
Mais de 9 anos	34 (47,9)	14 (19,7)	20 (28,2)		11 (15,5)	23 (32,4)	
Renda pessoal^†^				0,9			0,07
Até 1 SM	49 (70,0)	18 (25,7)	31 (44,3)		9 (12,9)	40 (57,1)	
> 1 SM	21 (30,0)	8 (11,4)	13 (11,4)		8 (11,4)	13 (18,6)	
Renda familiar				0,22			0,09
Até 1 SM	28 (40,0)	8 (11,4)	20 (28,6)		4 (5,7)	24 (34,3)	
> 1 SM	42 (60,0)	18 (25,7)	24 (34,3)		13 (18,6)	29 (41,4)	
Idade, anos				0,26			0,054
≥70	16 (22,5)	8 (11,3)	8 (11,3)		7 (9,8)	11 (15,5)	
<70	55 (77,5)	19 (26,8)	36 (50,7)		9 (12,7)	44 (62,0)	
Obstrução				0,018			0,28
Leve/ausente	50 (72,5)	23 (33,3)	27 (39,1)		14 (20,3)	34 (49,3)	
Moderada/grave	19 (27,5)	3 (4,3)	16 (23,2)		4 (5,8)	17 (24,6)	
Tabagismo				0,1			0,16
Não/ex-fumante	50 (70,5)	22 (31,0)	28 (39,5)		15 (21,1)	35 (49,3)	
Sim	21 (29,5)	5 (7,0)	16 (22,5)		3 (4,2)	18 (25,4)	

Resultados expressos por n (%).

* Grau de instrução completo ou não; ^†^ o SM brasileiro em 27 setembro de 2020 era de U$ 184.00.

ACT: *Asthma Control Test*; GINA: *Global Initiative for Asthma*; SM: salário mínimo.

Na tabela 3, vê-se a distribuição da amostra segundo suas respostas à pergunta-chave do estudo, e a concordância de cada item com o ACT e o GINA. Houve concordância entre a opinião do paciente sobre o controle da asma e o ACT em 49 (69,0%) casos; o coeficiente de Kappa entre a percepção do paciente do controle de sua doença e o nível de controle segundo o ACT foi 0,4, sendo considerado discreto. Houve concordância em 44 (62,0%) casos entre a opinião do paciente e o questionário proposto pelo GINA. O coeficiente de Kappa foi 0,29, também sendo considerado discreto. Entre os 34 participantes que assinalaram a opção bem controlada na questão-chave do estudo, 22 (64,7%) não estavam controlados segundo o documento GINA, e 17 (50%) não estavam controlados no próprio ACT.

**Tabela 3:** Distribuição da amostra segundo suas autoavaliações* do controle de asma e concordâncias em relação ao nível de controle no *Asthma Control Test* e na *Global Initiative for Asthma*

Percepção do paciente	ACT				GINA			
	
	Asma controlada	Asma não controlada	Total	Concordância	Asma controlada	Asma não controlada	Total	Concordância
Não controlada em absoluto	0	2 (2,8)	2 (2,8)	100	0	2 (2,8)	2 (2,8)	100
Mal controlada	0	7 (9,9)	7 (9,9)	100	0	7 (9,9)	7 (9,9)	100
Um pouco controlada	4 (5,6)	17 (23,9)	21 (29,5)	81	2 (2,8)	19 (26,8)	21 (29,6)	90,5
Bem controlada	17 (23,9)	17 (23,9)	34 (47,9)	50	12 (16,9)	22 (31,0)	34 (47,9)	35,3
Completamente controlada	6 (8,4)	1 (1,4)	7 (9,9)	85,7	4 (5,6)	3 (4,2)	7 (9,8)	57,1
Total	27 (38,0)	44 (62,0)	71 (100)	69	18 (25,3)	53 (74,7)	71 (100)	62

Resultados expressos por n (%) ou somente %.

* Definida pelas opções de respostas selecionadas pelos participantes a uma das perguntas do ACT, que são mostradas na primeira coluna da esquerda. Controlados assinalaram opções bem controlada ou completamente controlada. Não controlados assinalaram pouco controlada, pobremente controlada ou totalmente descontrolada.

ACT: *Asthma Control Test;* GINA: *Global Initiative for Asthma*.

Na tabela 4, separou-se a amostra em controlados (os que assinalaram na questão-chave totalmente controlado ou bem controlada) e não controlados (que assinalaram totalmente descontrolada, pobremente controlada ou um pouco controlada). Verifica-se que, utilizando-se o ACT, 43,9% dos pacientes não perceberam a falta de controle da asma e, ao se usar o GINA, esse percentual subiu para 61%. Considerando-se a má percepção do nível de controle em toda a amostra, 67% perceberam mal seu nível de controle.

**Tabela 4:** Percepção de controle de asma pelo paciente em relação ao *Asthma Control Test* e à *Global Initiative for Asthma*

Percepção de controle pelo paciente*	ACT			GINA			Total
	
	Controlados	Não controlados	Percepção inadequada	Controlados	Não controlados	Percepção inadequada	
Controlados	23 (32,5)	18 (25,3)	43,9	16 (22,5)	25 (35,2)	61,0	41 (47,7)
Não controlados	4 (5,6)	26 (36,5)	13,3	2 (2,8)	28 (39,5)	6,7	30 (42,2)
Total	27 (38,1)	44 (61,9)	57,2	18 (25,3)	53 (74,7)	67,7	71 (100)

Resultados expressos por n (%) ou somente %.

* Definida pela resposta ao ACT: “Como você avalia o controle da sua asma durante as últimas 4 semanas?”. Controlados assinalaram as opções bem controlada ou completamente controlada. Não controlados assinalaram pouco controlada, pobremente controlada ou totalmente descontrolada.

ACT: *Asthma Control Test*; GINA: *Global Initiative for Asthma.*

Na tabela 5, vê-se que a percepção do controle não teve relação estatisticamente significativa com o perfil sociodemográfico e clínico, independentemente do questionário utilizado, GINA ou ACT.

**Tabela 5:** Associação da qualidade da percepção de controle de asma com características sociodemográficas e clínicas

Variável	Percepção de controle pelo ACT*				Percepção de controle pela GINA*			
	
	Total	Correta	Incorreta	Valor de p	Total	Correta	Incorreta	Valor de p
Sexo				0,46				0,6
Feminino	57 (85,1)	41 (61,2)	16 (23,9)		59 (85,5)	36 (52,2)	23 (33,3)	
Masculino	10 (14,9)	8 (11,9)	2 (3,0)		10 (14,5)	6 (8,7)	4 (5,8)	
Estado civil				0,66				0,53
Casado	29 (43,3)	22 (32,8)	7 (10,5)		30 (43,5)	17 (24,6)	13 (18,9)	
Não casado	38 (56,7)	27 (40,3)	11 (16,4)		39 (56,5)	25 (36,2)	14 (20,3)	
Grau de instrução^†^				0,44				0,96
Até 9 anos	35 (52,2)	27 (40,3)	8 (11,9)		36 (52,1)	22 (31,8)	14 (20,3)	
Mais de 9 anos	32 (47,8)	22 (32,8)	10 (14,9)		33 (47,9)	20 (29,0)	13 (18,9)	
Renda pessoal^‡^				0,78				0,1
Até 1 SM	46 (69,7)	33 (50,0)	13 (19,7)		28 (70,6)	26 (38,2)	22 (32,4)	
>1 SM	20 (30,3)	15 (22,7)	5 (7,6)		20 (29,4)	15 (22,0)	5 (7,4)	
Renda familiar				0,44				0,14
Até 1 SM	28 (47,8)	19 (28,8)	9 (13,6)		28 (41,2)	14 (20,6)	14 (20,6)	
>1 SM	38 (52,8)	29 (44,0)	9 (13,6)		40 (58,8)	27 (39,7)	13 (19,1)	
Idade, anos				0,98				0,46
≥70	15 (22,4)	11 (16,4)	4 (7,5)		16 (23,2)	11 (15,9)	5 (7,3)	
<70	52 (77,6)	38 (56,7)	14 (20,9)		53 (76,8)	31 (44,9)	22 (31,9)	
Obstrução				0,28				0,65
Leve/ausente	45 (69,2)	35 (53,8)	10 (15,4)		36 (68,7)	28 (41,8)	18 (26,9)	
Moderada/grave	20 (30,8)	13 (20,0)	7 (10,8)		21 (31,3)	14 (20,9)	7 (10,4)	
Tabagismo				0,9				0,32
Não/ex-fumante	48 (71,6)	35 (52,2)	13 (19,4)		49 (68,7)	28 (40,6)	21 (30,4)	
Sim	19 (28,4)	14 (20,9)	5 (7,5)		20 (31,3)	14 (20,3)	6 (8,7)	

* Definida pela resposta ao ACT: “Como você avalia o controle da sua asma durante as últimas 4 semanas?”. Controlados assinalaram opções bem controlada ou completamente controlada. Não controlados assinalaram pouco controlada, pobremente controlada ou totalmente descontrolada; ^†^ grau de instrução completos ou não; ^‡^ o SM brasileiro em 27 setembro de 2020 era de U$ 184.00.

ACT: *Asthma Control Test*; GINA: *Global Initiative for Asthma*; SM: salário mínimo.

## DISCUSSÃO

Neste serviço, demonstra-se a técnica inalatória a todos os pacientes em todas as consultas. Solicita-se, invariavelmente, que os pacientes tragam seus dispositivos inalatórios vazios para treinamento em todas as consultas, e, quando o fazem, eles realizam a técnica inalatória diante do médico; quando não o trazem, os médicos sempre demonstram a técnica com um mecanismo vazio igual ao usado por cada paciente. Se constatados equívocos, estes são sempre corrigidos.^(12)^ Além disso, no ambulatório investigam-se e tratam-se as principais comorbidades relacionadas à asma, como rinite, refluxo gastresofágico, ansiedade, depressão e tabagismo. Talvez essas condutas sejam a justificativa para termos observado que 25% a 38% da amostra apresentava a asma controlada. Se é ainda abaixo do ideal, é maior que os 9% e 13% observados em estudos nacionais recentes,^(5,13)^ apesar de se manejarem os casos de mais alta complexidade neste ambulatório de hospital terciário. Ao se utilizar o ACT, não se observaram associações entre não controle e baixa renda ou escolaridade, como demonstrado anteriormente.^(14)^ Por outro lado, verificou-se que pacientes não casados e com obstruções mais graves mais frequentemente não estavam controlados, sugerindo que atenção especial deva ser dispensada a esses grupos. Em contrapartida, o GINA não demonstrou associações entre características clínicas, sociodemográficas ou funcionais da amostra e controle da asma, remetendo à ideia de que a supervisão do nível de controle deve ser universal.

A asma mal controlada ou incorretamente tratada pode evoluir desfavoravelmente com exacerbações quase fatais e até resultar em óbito.^(15)^ Até 66% dos pacientes com asma não informam espontaneamente os sintomas ao médico,^(16)^ e cerca de 50% com sintomas persistentes graves consideram sua doença bem controlada.^(17)^ A má percepção do controle da asma pelo paciente pode levar à avaliação inadequada da doença pelo médico e, consequentemente, a um tratamento insuficiente ou excessivo.^(16)^ Ainda não há consenso sobre um padrão-ouro para mensurar controle objetivamente.^(12)^ O documento atual do GINA enfatiza a necessidade de avaliar o controle da asma para orientar as decisões de manejo da asma, e propõe o uso de um questionário, que, apesar de amplamente recomendado e aceito, não é formalmente validado.^(4)^ O ACT^(11)^ é um questionário validado, inclusive para o português, e é reprodutível e sensível para a avaliação do controle.^(18)^ É uma das ferramentas recomendadas pela força-tarefa da *American Thoracic Society* (ATS)/*European Respiratory Society* (ERS) sobre a padronização das medidas de controle da asma.^(19)^Neste estudo, 41 (57,7%) percebiam-se controlados, quando, em verdade, 18 (43,9%) dos indivíduos não estavam controlados pelo ACT, e 25 (61%) não tinham controle pelo GINA. Estes resultados são inferiores aos 68,5% vistos em amostra diferente à deste estudo, pois se tratava de um inquérito realizado em farmácias,^(20)^ e superiores aos 27,2% obtidos quando se utilizou outro questionário, o *Asthma Control Questionnaire* (ACQ-6).^(13)^ Importante enfatizar que metade dos pacientes que assinalou especificamente a opção bem controlado no ACT falhou em perceber controle inadequado da asma segundo a pontuação obtida no próprio ACT, e, usando-se o GINA, 64,7% dos que marcaram essa opção, em realidade não tinham a doença controlada. Dentre os 30 pacientes que consideravam a asma não controlada, quatro (13,3%) a tinham controlada pelo ACT e dois (6,7%) segundo o GINA. Nesse último caso, a existência de outras comorbidades, como os distúrbios psiquiátricos, talvez seja a razão desse confundimento. Nesse ambulatório já foi verificado que, no mínimo, 53% dos pacientes têm ansiedade e/ou depressão.^(21)^ Talvez esses pacientes tenham alguma insatisfação com sua ventilação por somatizações ou hiperinsuflações, e tais fatos poderiam gerar repercussões matemáticas nas perguntas e nas pontuações em questionários. No total da casuística, 22 (31%) participantes interpretaram erradamente o nível de controle de sua doença quando se utilizou o ACT e 27 (38%) no GINA.

Outro aspecto a ressaltar é que, diferente de outros questionários, o ACT, em uma de suas questões, permite que o paciente faça uma autoavaliação de seu controle, que é a questão-chave deste estudo. Foi demonstrado que pacientes com má percepção de seu mau controle frequentemente respondem a essa questão de forma inadequada, assinalando equivocadamente as opções totalmente controlado ou, ainda mais frequentemente, a opção bem controlada. Isso aumenta a pontuação obtida nessa pergunta e, por conseguinte, no escore final do questionário, o que pode contribuir por classificá-lo como controlado no ACT – apesar de, por vezes, essa não ser a realidade. Utilizando-se, por exemplo, o questionário proposto no documento do GINA, que não oferece essa questão para autoavaliação, detectou-se um número ainda maior de pacientes não controlados, que se diziam controlados nessa casuística. De qualquer forma, o coeficiente de Kappa entre o controle da asma segundo o GINA ou o ACT, e a percepção de controle segundo o paciente foi discreto em ambos.

Não se confirmou o verificado previamente de que determinados grupos se percebem de forma inapropriada, como idosos,^(22)^ mulheres^(23)^ e portadores de asmas consideradas leves.^(21)^ Isso pode ter se dado em decorrência da exclusão realizada neste estudo dos pacientes controlados e sem percepção de seu controle adequado, visto o objetivo atual ser avaliar exclusivamente a percepção do mau controle. Nem o fato de ser um ambulatório coordenado por especialista parece influenciar na qualidade da percepção de controle dos pacientes.^(20)^

Dentre as limitações deste estudo, está a possibilidade de viés de seleção de pacientes que constituíram uma amostra de conveniência de um hospital de ensino, a qual pode não ser representativa da população geral de pacientes ambulatoriais. A recordação dos pacientes quanto aos sintomas de asma no último mês pode não ser totalmente precisa, não se podendo descartar a possibilidade de viés de memória. Outra limitação é o tamanho da amostra. Finalmente, a questão do ACT utilizada para avaliação da percepção dos pacientes sobre o controle da asma “Como você classificaria seu controle de asma nas últimas 4 semanas? não foi validada para esse fim.

Segundo nosso conhecimento, utilizando-se o ACT e o GINA, este é o único estudo observacional já realizado para investigar a dificuldade de percepção do mau controle da asma em hospital terciário em um estudo de vida real, no Brasil. A noção de controle parece pouco compreendida pelos pacientes asmáticos. Talvez estes se satisfaçam com o controle parcial da asma e considerem a persistência dos sintomas aceitável.^(24)^ Ouve-se com alguma frequência os pacientes proferirem frases como “Doutor, eu conheço o meu corpo”, mas esta percepção pode se demonstrar falha quando aferida por métodos objetivos. É preciso dobrar a atenção e a rigorosidade na avaliação do controle da asma. Além de investigar a adesão ao tratamento, a qualidade da técnica inalatória e o tratamento das comorbidades para obtenção do controle da asma, é importante lembrar que a má percepção do nível de controle é muito comum. Há pacientes que têm asma controlada e a consideram não controlada, mas, muito mais frequentemente, há pacientes que se consideram controlados sem, na verdade, esta ser a realidade. É necessária uma melhor educação quanto à importância, à percepção e aos métodos de identificação do controle. Talvez familiarizar os pacientes asmáticos com esses mesmos questionários e orientar a utilizá-los em suas residências sejam boas estratégias.

## CONCLUSÃO

Aplicando-se o *Asthma Control Test*, observou-se que quase metade dos participantes tinha percepção inadequada de seu mau controle da doença e, segundo o questionário do *Global Initiative for Asthma*, mais da metade da amostra não percebeu o descontrole da asma. A má percepção é muito comum, preocupante e impele à utilização de questionários na prática médica, para avaliação mais fidedigna do controle e da titulação correta do tratamento, independentemente das características dos pacientes.
